# Limited formal education is strongly associated with lower cognitive status, functional disability and frailty status in older adults

**DOI:** 10.1590/1980-57642018dn13-020011

**Published:** 2019

**Authors:** Allan Gustavo Brigola, Tiago da Silva Alexandre, Keika Inouye, Monica Sanches Yassuda, Sofia Cristina Iost Pavarini, Eneida Mioshi

**Affiliations:** 1 Nursing Post Graduate Program, Federal University of São Carlos, SP, Brazil.; 2 Department of Gerontology, Federal University of São Carlos, SP, Brazil.; 3 Gerontology, School of Arts, Sciences and Humanities, University of São Paulo, SP, Brazil.; 4 School of Health Sciences, University of East Anglia, Norwich, UK.

**Keywords:** cognition, instrumental activities of daily living, frailty, education, developing countries, cognição, atividades instrumentais da vida diária, fragilidade, educação, países em desenvolvimento

## Abstract

Limited formal education is still common in ageing populations. Although limited formal education seems to be independently and negatively associated with cognition, functional abilities and frailty in ageing, no studies have examined whether the gradient of limited formal education has an impact on health in later life.

**Objective:**

to examine the relationship of limited formal education with cognitive status, functional abilities, and frailty status.

**Methods:**

a cross-sectional study was conducted involving 540 older adults stratified into groups: no formal education, 12-24 months of education, and 25-48 months of education. Cognitive screening (MMSE), functional abilities (Lawton Index), and frailty (CHS criteria) were measured. Regression analyses were performed.

**Results:**

27% had no formal education, 21% had 12-24 months of formal education, and 55% had 25-48 months of formal education. Limited formal education has a clear gradient of negative impact: No formal education was associated with scoring below MMSE cut-off scores (OR = 7.9), being totally/partially dependent for IADLs (OR = 2.5) and frail (OR = 2.0). Having 12-24 months of education was associated with scoring below MMSE cut-off scores (OR = 5.2) and with being frail (OR = 2.0). The No formal education group was 10.1 times more likely to have worse cognitive scores, worse functional abilities and frailty/pre-frailty status concomitantly (CCoFF), while older adults who had 12-24 months of education had a 4.6 times greater chance of having CCoFF.

**Conclusion:**

limited education had a gradient association with cognitive performance, functional disability and frailty. These findings clearly emphasize the importance of prevention through education from childhood to older age.

## RESUMO.

A baixa escolaridade ainda é comum na população idosa. Embora a limitação na educação formal pareça estar independentemente e negativamente associada à cognição, habilidades funcionais e fragilidade no envelhecimento, nenhum estudo examinou a associação entre baixa escolaridade e um impacto futuro na saúde.

**Objetivo:**

esse estudo examinou a relação entre baixa escolaridade e o *status* cognitivo, habilidades funcionais e fragilidade.

**Métodos:**

estudo transversal com 540 idosos divididos em grupos: sem educação formal, 12-24 meses de escolaridade e 25-48 meses de escolaridade. Informações da triagem cognitiva (MEEM), habilidades funcionais (Índice de Lawton); a fragilidade (critérios do CHS) foram coletadas. Análises de regressão foram realizadas.

**Resultados:**

27% não tinham educação formal, 21% tinham entre 12-24 meses de educação formal e 55% tinham entre 25-48 meses de educação formal. Baixa escolaridade apresentou um impacto negativo e gradiente: nenhuma educação formal foi associada à pontuação abaixo do escore do MEEM (OR = 7,9), à dependência total/parcialmente em AIVD (OR = 2,5) e fragilidade (OR = 2,0). Ter 12-24 meses de escolaridade foi associado à pontuação abaixo do escore do MEEM (OR = 5,2) e a ser frágil (OR = 2,0). O grupo sem educação formal foi 10,1 vezes mais provável de apresentar piores escores cognitivos, pior capacidade funcional e fragilidade/pré-fragilidade concomitante (CCoFF), enquanto adultos idosos que tinham entre 12-24 meses de escolaridade tiveram 4,6 vezes maior chance de apresentar CCoFF.

**Conclusão:**

a baixa escolaridade apresentou associação com desempenho cognitivo, limitações funcionais e fragilidade. Os achados enfatizam claramente a importância da prevenção através da educação desde a infância à velhice.

Limited levels of formal education (LFE) are still common in ageing populations, particularly those of low-income and middle-income countries (LMICs). In Brazil, about 20% of older adults cannot read and write,^1^ while in China this figure reaches about 50% of their older adult population.^2^ In India, 70% of older adults have formal education that is well below primary school level.^3^ The Organisation for Economic Co-operation and Development (OECD) has stated that LFE in older adults is an area of major concern, as research has demonstrated its negative impact on life expectancy: older adults who complete 8-11 years of formal education are likely to have 1-17 years longer life expectancy compared to those without the same level of education.^4^ In parallel, socioeconomic status, often measured as a combination of education, income and occupation, has also been shown to influence overall functioning and independence during the ageing process.^5^ For this reason, it is critical to understand whether a gradient of low education can contribute to better cognition, functional abilities and frailty status in aging.

Length of formal education is also associated with poor performance on standardized cognitive tests. A South Korean study demonstrated that cognitive tests tend to be time-consuming and that reasons for marked difficulties in neuropsychological tests include poor comprehension, reading, and writing skills in those who are illiterate compared with high-educated older adults.^6^ In addition, a systematic review showed that illiteracy and little formal education seem to be strong factors determining dementia onset.^7^^-^ 9

Several recent studies have examined the impact of having limited formal education on functional abilities. Limited formal education seems to have a negative effect on instrumental activities of daily living (IADL) in older adults, as shown in studies conducted in the Netherlands and Brazil.^10^^,^ 11 A Mexican study has shown that every additional year of formal education leads to an improvement in ADL scores (0.06 points), as measured by the Katz index (score 0-5).^12^ The direct comparison of advanced and instrumental ADL performance in community-dwelling older adults with different levels of formal education has revealed that little or no formal education (≤4 years) were associated with significantly lower activity participation (e.g. engaging in social visits, going to church, housework, cooking and watching television) than those with higher levels of formal education in Brazil.^13^ However, less is known about the gradient of limited formal education effects on IADL, and whether older adults with low levels of formal education have greater difficulty performing IADL tasks or major limitations performing these activities.

Limited formal education can also be a predictor of frailty in older adults in Brazil.^14^^,^ 15 Frailty is a dynamic and multidimensional syndrome, that affects human functioning and is caused by a range of variables that increase the risk for dependency, institutionalization and death in old age.^16^^,^ 17 Older adults with low education in Europe also seem to have a three times higher risk of being categorized as frail, as opposed to older adults with higher levels of education.^18^ A Dutch study indicated that mature and older people who have completed higher education had consistently less overall frailty than people with primary or secondary education. In this study, the frailty components associated with low education were more morbidities, worse self-rated health, low psychosocial health and IADL limitations.^19^ Despite prior studies investigating education and frailty, no studies have examined whether a gradient of low levels of formal education (e.g. 12 months as opposed to 24 months in formal education) has an impact on frailty in old age.

Finally, the question of urban and rural settings also plays a role in limited formal education. There is still a higher prevalence of individuals with little or no formal education residing in rural than urban areas. Around the world, the educational system in urban settings is considered better compared to non-urban settings and, in addition, students who attend school in urban areas tend to perform better than those from rural areas.^20^

Although limited formal education seems to be independently and negatively associated with cognition, functional abilities and frailty in ageing, to the best of our knowledge, no studies have investigated whether a gradient of formal education can lead to different health outcomes. This gradient is important because a slightly longer period spent in formal education may be a potential protective factor for health outcomes in old age. In addition, examining these three key variables in the same s study is of great relevance. The condition of presenting with these three adverse health outcomes (poor cognition, functional dependence and frailty) may portray a context of vulnerability of older adults; this operationalization, however, has not been investigated to date. Moreover, the literature includes many studies which have examined the influence of education on at least one of these key variables. However, most of these studies were conducted in high-income countries, where limited formal education tends to be less common in old age. Thus, the present paper aimed to address this gap by examining the relationship of limited formal education with these three key factors in a healthy ageing sample. Our hypotheses were that there are associations between low education levels and health outcomes (i.e. negative influence on frailty and function), residential setting (i.e. rural residents have worse outcomes) and relationship status (i.e. having a partner is protective).

## METHODS

This is a secondary analysis of a larger study conducted in community-dwelling older adults from São Carlos, Brazil. São Carlos is a medium-sized town in Brazil, located in the state of Sao Paulo. The town population comprises approximately 222,000 inhabitants, where 13% of the population is aged 60 years or older.^21^

### Participants

This study is part of “The variables associated with cognition in older adults caregivers” study run by the Ageing and Health Research Group at the Federal University of São Carlos, Brazil.

The main study has been described elsewhere,^22^ but a brief description follows. All community-dwelling older adults (age ≥60 in Brazil, as defined by the World Health Organization) residents registered at 18 primary health care centres (n = 1,188) in São Carlos, Brazil, were contacted in-person and invited to participate in a survey. The response rate was 59.1% and 702 older adults of all educational levels participated in the study. For the present study, 540 were included in the analysis and the sole criterion for entry was limited formal education, as defined by 0-4 years of formal schooling.

Interviews were conducted by trained research assistants (AB; BL; MT; ER; NO; EN)^23^ at the participants’ homes, and interviews lasted between 1-2 hours. The assistants were professionals in Nursing and Gerontology fields. Interviews were conducted between April and November 2014.

### Assessments and instruments

A questionnaire assessment proforma including key demographic variables such assex (male, female), age (in years), marital status (living with someone in a marital-like relationship), occupation (retired/have pension, retired and have part-time/casual work, employed full-time, doing unpaid work/unemployed), residential setting (urban, rural) and self-declared number of years in formal education is presented in Table 1.

**Cognitive screening:** The MMSE is a brief cognitive assessment used in clinical and research settings worldwide. Scores range from 0-30, with lower scores denoting impairment in cognitive function. The MMSE evaluates orientation (place and time), memory, attention and calculation, language (written, reading, command, repetition and naming) and visuo-constructional abilities (a = 0.765). Scores on the MMSE were analysed utilizing cut-offs for different levels of formal education published previously and used in Brazil: participants with 1-4 years of formal education had a cut-off of 22/30, and those without formal education had a cut-off of 17/30.^24^ Scores below cut-off suggest cognitive impairment.
  **Table 1:** Participants’ demographic characteristics and scores on cognition, activities of daily living, and frailty status, stratified by level of formal education in months (n = 540). São Carlos, Brazil, 2014.

		Total (N = 540)	No education (n = 145)^a^	12-24 months of education (n = 113)^b^	25-48 months of education (n = 282)^c^
Age (min 60; max 98), Means (95 CI)^±1^		72.1 (71.4-72.7)	75.5 (74.0-77.0)	70.9 (69.5-72.3)	71.0 (69.9-71.6)
Sex (male), % ^#1^		44.1	42.8	42.5	45.4
Setting (rural), % ^#2^		75.0	89.0 (0.3: 0.2-0.5)	69.0 (1.0: 0.6-1.7)	70.2 (1.0)
Living with someone in marital-like relationship, %^#3^		89.1	82.1 (2.3: 1.3-4.3)	92.0 (0.9: 0.4-2.1)	91.5 (1.0)
Occupation, %	Retired	63.9	68.3	54.0	65.6
	Retired with casual work	12.0	11.7	10.6	12.8
	In full-time work	5.6	2.8	5.3	7.1
	Doing unpaid work	18.5	17.2	30.1	14.5
Cognition: MMSE (max score 30) Means (95 CI)^±2^		21.00 (20.6-21.4)	17.0 (16.3-17.9)	20.1 (19.3-21.0)	23.3 (22.9-23.8)
Proportion of older adults who scored below cut-off (MMSE), %		44.3	73.1	60.2	23.0
IADL: Lawton & Brody Index (max score 21) Means (95 CI) ^±3^		16.6 (16.2-16.9)	14.6 (13.9-15.3)	17.4 (16.7-18.0)	17.2 (16.8-17.7)
IADLs: totally dependent, %		4.3	6.49	2.7	3.5
IADLs: partially dependent, %		75.0	82.1	75.2	71.3
Frailty criteria (n of factors max 5) Means (95 CI)^±4^		1.7 (1.6-1.8)	2.2 (1.9-2.4)	1.7 (1.4-1.9)	1.5 (1.4-1.8)
Frail, %		27.6	40.0	29.2	20.6
Pre-frail, %		52.2	46.2	52.2	55.3
Non-frail, %		20.2	13.8	18.6	24.1

± ANOVA one-way:

±1 F = 19.2; p < 0.01; a≠b, a≠c, b = c.

±2 F = 104.1; p < 0.01; a≠b, a≠c, b≠c.

±3 F = 23.8; p < 0.01;a≠b, a≠c, b = c.

±4 F = 11.5; p < 0.01; a≠b, a≠c, b = c.

# Pearson Chi-square test.

#1 Stat = 0.4; p = 0.812; a = b, a = c, c = b.

#2 Stat = 20.6; p < 0.01; a≠b, a≠c, b = c.

#3 Stat = 10.0; p < 0.01; a≠b, a≠c, b = c.

MMSE: Mini-Mental State Examination. IADLs: instrumental activities of daily living.
**Functional abilities:** Functional abilities were evaluated with the IADL Lawton and Brody Index,^25^ which assesses the degree of independence for the following instrumental ADLs: use of telephone, travelling to places, shopping, preparing meals, performing housework tasks, taking medications and managing finances (a = 0.843). For each activity, an informant rates the level of dependence of the elder on the task (3 = does not need assistance; 2 = needs partial assistance; 1 = needs total assistance). Scores range from 7-21, where 21 represents total independence; 8-20 partial dependence, and < = 7 points reflect total dependence. For the statistical analyses, total and partial dependence were combined into one category.
**Frailty:** Frailty was defined using Fried’s phenotype,^26^ which includes (1) unintentional weight loss in the last year, (2) exhaustion in the last week, (3) muscular weakness, (4) slowness, and (5) decreased physical activity level compared to the previous year.

*Unintentional weight loss* was evaluated by the question “In the last twelve months, did you lose weight in the absence of dieting?”. The cut-off is defined as weight loss >4.5 kg or >5% of body weight.^26^ *Exhaustion* was assessed by two items from the depression scale (Center for Epidemiological Studies - Depression, CES-D):^27^^,^ 28 “I felt that everything I did was an effort?” and “I could not get going”. *Muscular weakness* was indicated by the average of three consecutive measurements of grip strength of the dominant hand in kilograms force using a Jamar hydraulic dynamometer (model SH5001, SAEHAN®, Lafayette, Illinois, USA). Results were adjusted for sex and Body Mass Index (BMI).^29^ *Slowness* was evaluated using the average of three consecutive measurements of time (in seconds) that the participant took to walk 4.6 meters (straight line, even surface, normal pace, and using an assistive device if normally needed). To enable acceleration and de-acceleration, two meters were added at the beginning and end of the route, totalling an 8.6 meter walk. Results were adjusted for gender and height. *Low level of physical activity* was indicated by an affirmative answer to the question: “Do you think you do less physical activity than twelve months ago?”.

Utilizing Fried’s model, participants were categorized into different levels of frailty: frail (3-5 criteria), pre-frail (1-2 criteria) and non-frail/robust (negative responses on all five criteria).^26^

### Ethics approval

This project was authorized by the Department of Health, São Carlos Cityand approved bytheEthics Committee on Human Research, Federal University of São Carlos. All participants gave their written consent.

### Data analyses

We stratified the sample into different levels of limited formal education for comparison between groups (*no time spent in formal education; 12-24 months of education; 25-48 months of education*). Descriptive analyses including proportion (%), mean and dispersion (95 CI = 95% Confidence Interval) were performed for each group. First, one-way ANOVA with Tukey post hoc tests were performed to compare differences in age (continuous) between the education groups. Chi-square tests, including odds ratio (OR) statistics, were performed to analyse associations between limited formal education and sex (male, female), residential setting (urban, rural) and living with someone in a marital-like relationship (yes, no).

Analyses, including binary logistic and multinomial regressions, were performed to investigate associations between educational levels and the health outcomes, controlled for age (>74 years), sex (male) and residential setting (rural). The control variables were included in multivariate regressions models if exhibiting associations with *p-value<*0.2 on univariate binary logistic regressions with the three health outcomes.

The association between low educational levels and cognition (below MMSE cut-off - category tested; above MMSE cut-off - reference category) and the association between low educational levels and functional abilities (partially/totally dependence - category tested; no IADL impaired - reference category) were tested using binary regressions. Multinomial regressions were performed to test the association between low educational levels and frailty status (pre-frailty - category tested; frailty - category tested; non-frailty - reference category). For these comparisons, the groups *No education* and *12-24 months of education* were entered in models with the highest education group (*25-48 months of education*) serving as reference (Tables 2-4).

**Table 2:** Factors (age, sex, setting and level of formal education) associated with scoring below cognitive cut-off (MMSE). Odds ratio, 95% confidence intervals in brackets.

Variables	MMSE
	Below cut-off (MMSE)
Age >74y	1.6 (1.1-2.5)*
Males	0.8 (0.5-1.3)
Rural setting	0.7 (0.4-1.3)
No formal education	7.9 (4.9-12.6)*
12-24 months of education	5.2 (3.2-8.3)*

Categories/groups of references in the model: Above cut-off (MMSE), Age 60-74y, Females, Urban setting, 25-48 months of education.

* p < 0.05.

**Table 3:** Factors (age, sex, setting and level of formal education) associated with being categorized as totally dependent for IADLs and partially dependent (Lawton & Brody Index) Odds ratio, 95% confidence intervals in brackets.

Variables	Lawton & Brody Index
	IADL partially/totally dependent
Age >74y	2.3 (1.3-4.0)*
Males	5.3 (3.0-9.2)**
Rural setting	0.4 (0.5-1.6)
No formal education	2.5 (1.3-4.7)**
12-24 months of education	1.2 (0.7-2.1)

Categories/groups of references in the model: Non IADL impaired, Age 60-74y, Females, Urban setting, 25-48 months of education

* p < 0.05.

** p < 0.01.

**Table 4:** Factors (age, sex, setting and level of formal education) associated with being categorized as frail and pre-frail. Odds ratio, 95% confidence intervals in brackets.

Variables	Frailty groups	
	Frail	Pre-frail
Age >74y	7.3 (3.4-15.4)**	4.0 (2.0-7.9)**
Males	0.4 (0.2-8)*	0.6 (0.3-1.0)
Rural setting	0.2 (0.1-0.5)**	0.4 (0.2-0.7)**
No formal education	2.0 (1.0-3.9)*	1.0 (0.5-1.9)
12-24 months of education	2.0 (1.0-4.1)*	1.3 (0.7-2.4)

Categories/groups of references in the models: Non-frail, Age 60-74y, Females, Urban setting, 25-48 months of education.

* p < 0.05.

** p < 0.01.

To investigate whether the older adults with *no formal education* and *12-24 months of formal education* had greater risk of presenting the three adverse conditions together (**C**oncomitant worse **Co**gnitive scores, worse **F**unctional abilities, and pre-**F**railty or **F**railty - CCoFF), CCoFF was entered as a dependent variable into regression models. For these analyses, age (>74 years), sex (male), and residential setting (rural) were control variables. The groups *No education* and *12-24 months of education* were entered in models with the highest education group (*25-48 months of education*) used as reference (Table 5).

**Table 5:** Factors (age, sex, setting and level of formal education) associated with CCoFF status. Odds ratio, 95% confidence intervals in brackets.

Variables	CCoFF condition
	Concomitant below cut-off (MMSE), at least one IADL dependence and pre-frailty or frailty (CCoFF)
Age >74y	3.3 (1.6-6.9)*
Males	3.7 (1.7-7.7)*
Rural setting	0.7 (0.3-1.4)
No formal education	10.1 (4.3-23.6)**
12-24 months of education	4.6 (2.2-9.8)*

Significance level was set at *p*≤0.05, 95% Confidence Interval (95%CI). All analyses were performed using the Statistical Package for the Social Sciences (SPSS) version 21.0 (IBM Inc., Chicago, Illinois, USA).

## RESULTS

Participant demographic characteristics are shown in Table 1. Forty-four percent of the participants were male, with average age of 72 years (range 60-98). Seventy-five percent of the participants were urban dwellers, 89% were married or living with a partner, and 64% were retired. The group with *No formal education* was older when compared to the other two groups (*12-24 months of education* and *25-48 months of education; one-way* ANOVA: F = 19.2; *p* < 0.01). The *No formal education* group was more likely to live in urban settings (Chi-square test: *p* < 0.01), and to be living with a partner (Chi-square test: *p* < 0.01), in comparison to the other groups with more years of formal education. Overall, the groups with *12-24 months of education*, and *25-48 months of education* were similar in relation to residential setting and relationship status (Table 1).

### What is the impact of low formal education on cognitive status?

Table 2 shows that the *No formal education* group was 7.9 times more likely to score below the cut-off of the MMSE, independent of age, sex or place of residence. The group with *12-24 months of education* had 5.2 times more chance of scoring below the cut-off; in addition, being older than 74 years of age also increased the chances of scoring below the cut-off on the MMSE by 1.6 times (Table 2).

### What is the impact of low formal education on functional abilities?

The *No formal education* group was 2.5 times more likely to have total/partial IADL dependence, irrespective of age, sex or residential setting (Table 3). Having *12-24 months of education* was not associated with total/partial dependence for IADLs. A secondary finding was that older age was associated with dependence, where being male was the strongest factor associated with this outcome (Table 3). The category of reference for the dependent variable was the non-IADL impaired/IADL independent group.

### What is the impact of low formal education on frailty?

Having *No formal education* increased the chance of being categorized as frail by 2.0 times. A similar magnitude of association was found between *12-24 months of education* and *frailty* (OR = 2.0) (Table 4). Belonging to either of the low education groups did not increase the chance of being categorized as pre-frail.

In addition, age (>74 years) increased the chances of being frail and pre -frail. Being male was inversely associated with frailty, while living in rural settings was a protective factor in frailty and pre-frailty (Table 4). For these analyses, the category of reference for the dependent variable was the non-frail older adults group.

### What are the characteristics of the older adults who have Concomitant worse Cognitive scores, worse Functional abilities, and pre-Frailty/Frailty (CCoFF)?

The proportion of older adults who had CCoFF was 33.8% (183 participants). The prevalence of CCoFF in the *No formal education* group was 15.5% (n = 84), in the *12-24 months of education* was 9.6% (n = 52) and in 25-48 months of education was 8.7% (n = 47). Participants in the *No formal education* group were 10.1 times more likely to present CCoFF (see Table 5), while participants with *12-24 months of education* had 4.6 times more chance of having CCoFF. Being older than 74 years and being male were associated with having CCoFF, with similar magnitudes of association (OR = 3.3 and OR = 3.7, respectively).

## DISCUSSION

This study demonstrated that low levels of education (or the absence of formal education) had a gradient of negative impact on the older adults: limited formal education had greatest adverse impact on cognitive status, followed by negative association with functional abilities and a lesser effect in pre-frailty. In other words, more years of formal education were directly associated with better scores on brief cognitive tests, enhanced functional abilities and lower frailty.

The negative influence of limited formal education on cognitive scores has been previously described, and our results corroborated similar findings in Brazil, India and China.^30^^-^ 32 Performance on neuropsychological tests may be influenced by intellectual and communication skills, abilities developed during the schooling period. Thus, older adults with limited formal education tend to have lower scores on many usual tests compared to high-educated older adults. This is an important issue because diagnosing cognitive disorders in low-educated older adults could prove more complex and difficult. In our study, even belonging to the group with little formal education (12-24 months) already yielded better cognitive performance on classic instruments of cognitive examination, such as the MMSE.

With regards to function, as measured by IADL assessments, only the *No formal education* group had a negative association with total/partial dependence in our study (OR = 2.5; 95% CI 1.3-4.7; *p* < 0.01); whereas the *12 to 24 months of education* did not (OR = 1.2; 95% CI 0.7-2.1; *p* = 0.48). It seems that age remains a very important factor in functional dependence. A large Brazilian study reported that older adults with no formal education and individuals with limited formal education had a great number of disabilities,^33^ a finding that is analogous to that of the present study. Additionally, another study reported that education is a strong factor influencing functional limitations in chronic conditions, such as diabetes.^34^

The specific reasons behind the link between limited formal education and decreased functional abilities are not entirely clear, but the contribution of cognition to IADLs cannot be understated. Complex ADLs require high cognitive skills, which can be negatively influenced by poor cognitive performance.^35^ Other possible reasons include the fact that limited formal education can lead to barriers in communication, which creates difficulties for engagement in more complex activities in the community and at home. A Japanese study suggested that engaging in paid work can be a protective factor for decline in IADL among older adults, but finding a job depends on educational level during life.^36^ Reasons for the paucity of studies in this area are likely to be related to the low number of research studies in developing countries, where there is a higher number of older adults with limited formal education.

In the present study, no direct association was found between limited formal education and pre-frailty (*No formal education*: OR = 1.0; 95% CI 0.5-1.9; *p* = 0.87; *12-24 months of education*: OR = 1.3; 95% CI 0.7-2.4; *p* = 0.32). Frailty status, however, was influenced by limited formal education in both groups (*No formal education:* OR = 2.0; 95% CI 1.0-3.9; *p* < 0.05; *12-24 months of education:* OR = 2.0; 95% CI 1.0-4.1; *p* < 0.05), while older age and rural setting were protective factors. A previous study showed that frailty in older adults was strongly dependent on levels of education.^14^ Reasons underpinning this vulnerability may include malnourishment in this group, for whom greater risk of weight loss,^37^ hip fractures and subsequent immobility,^38^ poor health habits and increased related comorbidity may be observed.^39^ These relationships could be explained by life-long low-income, limited access to information and services (e.g., health advice), poor housing conditions, unfavourable environment for development (e.g., pollution, violence), inadequate nutrition, and comorbidities. Evidence, therefore, suggests that higher levels of education could be the key to better health. Moreover, many conditions could be prevented, or have their negative effects reduced during the life course, if the risks of frailty in old age were to be reduced. Higher levels of formal education, reflecting ability to obtain information about healthy habits, was associated with high levels of non-frailty in Japan.^40^ Good quality of life and well-being, healthy behaviour that involves better physical activity, diet, substance use and medication, high social participation, no or mild cognitive or functional impairment, little or no disability, no or only few chronic diseases, survival to a specific age in good health and finally, autonomy in instrumental activities of daily living, have been described as components of healthy ageing.^41^ The education system in urban areas may have strengths compared to the system in rural settings and, consequently, might influence individuals’ health outcomes. In this study, living in a rural setting proved a protective factor for frailty. This could be explained by the pace of life style, less stress and good habits common in rural community-dwellers.^42^ However it is important to ascertain where the older adults attained their educational level and where they have lived for most of their life span. In particular, older adults residing in rural communities in Brazil seem to have better quality of life, independently of educational status and income. This can be explained by the simple rural lifestyle, where many resources are self-provided and good health habits (e.g. walking, plant-based diet) are present.^15^

In this study, limited formal education had a cumulative negative association in participants who had CCoFF (**C**oncomitant worse **Co**gnitive scores, worse **F**unctional abilities, and pre-**F**railty or **F**railty), with greater odds ratio compared to when each condition was analysed individually (*No formal education:* OR = 10.0; 95% CI 4.3-23.6; *p* < 0.01. *; 12-24 months of education:* OR = 4.6; 95% CI 2.2-9.8; *p* < 0.05). No other studies similar to the present investigation were identified. Cognitive frailty theory could explain our findings, in particular in relation to health style and environmental factors. Cognitive frailty is considered a syndrome, with pathological mechanisms that include cardiovascular disease, nutritional and hormonal dysregulation, inflammation, and a strong influence of health style and environmental context.^43^^,^ 44 Living with limited formal education is one of the multiple factors involving environment and lifestyle, where a clear gradient of limited formal education interacts gradually with worse cognition and frailty status, as well as more marked functional disability. The present study suggests that low levels of education can represent a risk for future vulnerability of older adults due to the strong association with CCoFF. Having high levels of education may sustain better life satisfaction and prevent health issues. Formal schooling is likely to support the development of good communication and problem-solving skills for life. These components can be useful across life events and could be key to good health in old age.

This study is not without limitations. We were not able to provide a clinical evaluation of pre-clinical dementia during this study, which limits some of the interpretations. A second limitation is our inability to stratify the groups of formal education by when they received their formal education, which could have other implications for the interpretation of our results.

Implications of these findings include the pressing need to test educational programs for older adults^45^ in order to elicit potential health benefits and reduce disability in these populations with limited formal education,^46^ especially in LMIC countries. More specifically, in Brazil, educational programs for mature and older adults are part of the national agenda, which would directly address the benefits identified in our study. These programmes would be equally relevant in areas of marked deprivation in high-income countries.

Research has shown that educational health interventions to improve the health profile of older adults is viable,^47^ but little is known about the effect of formal education on adherence to such health interventions and its influence on outcomes. Additionally, further studies could investigate the association of low education (and potentially associated deprivation) on access to information and services, housing, healthy environments and nutrition. Finally, these findings clearly highlight the importance of promoting formal education from childhood to older age.

## References

[1] Pilger C, Menon MH, de Freitas Mathias TA (2011). Socio-demographic and health characteristics of elderly
individuals: Support for health services. Rev Lat Am Enfermagem [Internet].

[2] Lei X, Strauss J, Tian M, Zhao Y (2011). Living arrangements of the elderly in China: evidence from the
CHARLS national baseline. China Econ J.

[3] Tripathi R, Kumar K (2012). Illiteracy and Cognition in Older Adults. Indian J Psychol Med.

[4] OECD (2013). Education indicator in focus. OECD Indicator.

[5] Kim D-S, Jeon G-S, Jang S-N (2010). Socioeconomic status, social support and self-rated health among
lone mothers in South Korea. Int J Public Health.

[6] Shim Y, Ryu HJ, Lee DW, Lee J-Y, Jeong JH, Choi SH (2015). Literacy Independent Cognitive Assessment: Assessing Mild
Cognitive Impairment in Older Adults with Low Literacy
Skills. Psychiatry Investig.

[7] Beydoun M, Beydoun H, Gamaldo A, Teel A, Zonderman A, Wang Y (2014). Epidemiologic studies of modifiable factors associated with
cognition and dementia: systematic review and meta-analysis. BMC Public Health [Internet].

[8] Prince M, Acosta D, Ferri CP, Guerra M, Huang Y, Llibre Rodriguez JJ (2012). Dementia incidence and mortality in middle-income countries, and
associations with indicators of cognitive reserve: a 10/66 Dementia Research
Group population-based cohort study. Lancet.

[9] Ferri CP, Acosta D, Guerra M, Huang Y, Llibre-Rodriguez JJ, Salas A (2012). Socioeconomic factors and all cause and cause-specific mortality
among older people in Latin America, India, and China: a population-based
cohort study. PLoS Med.

[10] Torres JL, Dias RC, Ferreira FR, Macinko J, Lima-Costa MF (2014). Functional performance and social relations among the elderly in
Greater Metropolitan Belo Horizonte, Minas Gerais State, Brazil: a
population-based epidemiological study. Cad Saude Publica [Internet].

[11] Koster A, Penninx BWJH, Bosma H, Kempen GIJM, Harris TB, Newman AB (2005). Is there a biomedical explanation for socioeconomic differences
in incident mobility limitation?. J Gerontol A Biol Sci Med Sci.

[12] Díaz-Venegas C, Wong R (2016). Trajectories of limitations in activities of daily living among
older adults in Mexico, 2001-2012. Disabil Health J.

[13] Sposito G, Neri AL, Yassuda MS (2016). Advanced Activities of Daily Living (AADLs) and cognitive
performance in community-dwelling elderly persons: Data from the FIBRA Study
- UNICAMP [Internet]. Rev Bras Geriatr Gerontol.

[14] Brigola AG, Rossetti ES, Santos BR, Neri AL, Zazzetta MS, Inouye K (2015). Relationship between cognition and frailty in elderly A
systematic review. Dement Neuropsychol.

[15] Brigola AG, Luchesi BM, Alexandre T da S, Inouye K, Mioshi E, Pavarini SCI (2017). High burden and frailty: association with poor cognitive
performance in older caregivers living in rural areas. Trends Psychiatry Psychother.

[16] Morley JE, Vellas B, van Kan GA, Anker SD, Bauer JM, Bernabei R (2013). Frailty consensus: a call to action. J Am Med Dir Assoc.

[17] Jesus ITM, Orlando F de S, Zazzetta MS (2018). Frailty and cognitive performance of elderly in the context of
social vulnerability. Dement Neuropsychol [Internet].

[18] Hoogendijk EO, van Hout HPJ, Heymans MW, van der Horst HE, Frijters DHM, Broese van Groenou MI (2014). Explaining the association between educational level and frailty
in older adults: results from a 13-year longitudinal study in the
Netherlands. Ann Epidemiol.

[19] Franse CB, van Grieken A, Qin L, Melis RJF, Rietjens JAC, Raat H, Abete P (2017). Socioeconomic inequalities in frailty and frailty components
among community-dwelling older citizens. PLoS One [Internet].

[20] OECD (2013). What makes urban schools different?.

[21] Instituto Brasileiro de Geografia e Estatística (2013). Diretoria de Pesquisas, Coordenação de Trabalho e Rendimento, Pesquisa
Nacional por Amostra de Domicílios.

[22] Luchesi BM, Alexandre T da S, Oliveira NA, Brigola AG, Kusumota L, Pavarini SCI (2016). Factors associated with attitudes toward the elderly in a sample
of elderly caregivers. Int Psychogeriatr.

[23] Pavarini SCI, Neri AL, Brigola AG, Ottaviani AC, Souza EN, Rossetti ES (2017). Elderly caregivers living in urban, rural and high social
vulnerability contexts. Rev Esc Enferm USP.

[24] Brucki SMD, Nitrini R, Caramelli P, Bertolucci PHF, Okamoto IH (2003). Suggestions for utilization of the mini-mental state examination
in Brazil. Arq Neuropsiquiatr.

[25] Lawton MP, Brody EM (1969). Assessment of older people: self-maintaining and instrumental
activities of daily living. Gerontologist.

[26] Fried LP, Tangen CM, Walston J, Newman AB, Hirsch C, Gottdiener J (2001). Frailty in older adults: evidence for a phenotype. J Gerontol A Biol Sci Med Sci.

[27] Radloff LS (1977). The CES-D Scale: a self report depression scale for research in
the general population. Appl Psychol Meas.

[28] Batistoni SST, Neri AL, Cupertino APFB (2007). [Validity of the Center for Epidemiological Studies Depression
Scale among Brazilian elderly]. Rev Saude Publica.

[29] Zortea K, Silva MLB (2011). Índice de massa corporal no adulto e no idoso. Arq Bras Cardiol.

[30] Amaral-Carvalho V, Caramelli P (2012). Normative Data for Healthy Middle-Aged and Elderly Performance on
the Addenbrooke Cognitive Examination-Revised. Cogn Behav Neurol.

[31] Mathuranath PS, Cherian JP, Mathew R, George A, Alexander A, Sarma SP (2007). Mini mental state examination and the Addenbrooke's cognitive
examination: effect of education and norms for a multicultural
population. Neurol India.

[32] Zhou S, Zhu J, Zhang N, Wang B, Li T, Lv X (2014). The Influence of Education on Chinese Version of Montreal
Cognitive Assessment in Detecting Amnesic Mild Cognitive Impairment among
Older People in a Beijing Rural Community. Sci World J.

[33] Ferreira PC dos S, Tavares DMS, Rodrigues RAP (2011). Características sociodemográficas, capacidade funcional e
morbidades entre idosos com e sem declínio cognitivo. ACTA Paul Enferm.

[34] Herd P, Goesling B, House JS (2007). Socioeconomic position and health: the differential effects of
education versus income on the onset versus progression of health
problems. J Health Soc Behav.

[35] Pereira FS, Yassuda MS, Oliveira AM, Forlenza O V (2008). Executive dysfunction correlates with impaired functional status
in older adults with varying degrees of cognitive impairment. Int psychogeriatrics.

[36] Fujiwara Y, Shinkai S, Kobayashi E, Minami U, Suzuki H, Yoshida H (2016). Engagement in paid work as a protective predictor of basic
activities of daily living disability in Japanese urban and rural
community-dwelling elderly residents: An 8-year prospective
study. Geriatr Gerontol Int.

[37] Majumder M, Saha I, Chaudhuri D (2014). Assessment of nutritional risk in community-dwelling older adults
(65 to 75 years) in Kolkata, India. J Nutr Gerontol Geriatr.

[38] Benetou V, Orfanos P, Feskanich D, Michaelsson K, Pettersson-Kymmer U, Ahmed LA (2015). Education, marital status, and risk of hip fractures in older men
and women: the CHANCES project. Osteoporos Int.

[39] Nordahl H, Osler M, Frederiksen BL, Andersen I, Prescott E, Overvad K (2014). Combined effects of socioeconomic position, smoking, and
hypertension on risk of ischemic and hemorrhagic stroke. Stroke.

[40] Shirooka H, Nishiguchi S, Fukutani N, Adachi D, Tashiro Y, Hotta T (2017). Association between comprehensive health literacy and frailty
level in community-dwelling older adults: A cross-sectional study in
Japan. Geriatr Gerontol Int.

[41] Fuchs J, Scheidt-Nave C, Hinrichs T, Mergenthaler A, Stein J, Riedel-Heller SG (2013). Indicators for Healthy Ageing - A Debate. Int J Environ Res Public Health [Internet].

[42] Monette M (2012). Rural life hardly healthier. CMAJ.

[43] Fougere B, Delrieu J, Del Campo N, Soriano G, Sourdet S, Vellas B (2017). Cognitive Frailty: Mechanisms, Tools to Measure, Prevention and
Controversy. Clin Geriatr Med.

[44] Ruan Q, Yu Z, Chen M, Bao Z, Li J, He W (2015). Cognitive frailty, a novel target for the prevention of elderly
dependency. Ageing Res Rev.

[45] Santos BR, Pavarini SCI, Brigola AG, Orlandi FS, Inouye K (2014). Factors associated with quality of life in elderly undertaking
literacy programs. Dement Neuropsychol.

[46] Dannefer D (2003). Cumulative Advantage/Disadvantage and the Life Course:
Cross-Fertilizing Age and Social Science Theory. Journals Gerontol Ser B [Internet].

[47] Costa-de Lima K, Peixoto-Veras R, Pereira-Caldas C, Motta L-B da, Bonfada D, Marques-Dos Santos M (2015). Effectiveness of intervention programs in primary care for the
robust elderly. Salud Publica Mex.

